# Serum Wisteria Floribunda Agglutinin-Positive Mac-2 Binding Protein Could Not Always Predict Early Cirrhosis in Non-Viral Liver Diseases

**DOI:** 10.3390/diseases4040038

**Published:** 2016-12-14

**Authors:** Yuki Haga, Tatsuo Kanda, Reina Sasaki, Masato Nakamura, Koji Takahashi, Shuang Wu, Shin Yasui, Makoto Arai, Shingo Nakamoto, Osamu Yokosuka

**Affiliations:** 1 Department of Gastroenterology and Nephrology, Graduate School of Medicine, Chiba University, 1-8-1 Inohana, Chuo-ku, Chiba 260-8677, Japan; hagayuki@gmail.com (Y.H.); reina_sasaki_0925@yahoo.co.jp (R.S.); nkmr.chiba@gmail.com (M.N.); koji517@gmail.com (K.T.); gosyou100@yahoo.co.jp (S.W.); ntcph863@yahoo.co.jp (S.Y.); araim-cib@umin.ac.jp (M.A.); nakamotoer@yahoo.co.jp (S.N.); yokosuka-osamu@funabashi.jcho.go.jp (O.Y.); 2 Japan Community Health care Organization Funabashi Central Hospital, 6-13-10 Kaijin, Funabashi, Chiba 273-8556, Japan

**Keywords:** autoimmune hepatitis, nonalcoholic steatohepatitis, primary biliary cholangitis, WFA(+)-M2BP, M2BPGi

## Abstract

Background: Wisteria floribunda agglutinin-positive human Mac-2-binding protein (WFA(+)-M2BP) is a novel non-invasive marker of liver fibrosis. The goal of the study was to investigate whether the novel serum biomarker WFA(+)-M2BP or other non-invasive markers are useful for the prediction of liver fibrosis in patients with nonalcoholic steatohepatitis (NASH), autoimmune hepatitis (AIH), and primary biliary cholangitis (PBC). Methods: We examined a significant correlation between serum WFA(+)-M2BP levels and histological staging of fibrosis in several chronic liver diseases, such as NASH, AIH, and PBC. Results: WFA(+)-M2BP could not predict hepatic fibrosis in these patients. We also showed that the level of platelet counts is a useful predictor of hepatic fibrosis progression in patients with NASH, AIH, and PBC. There was a significant correlation between staging of fibrosis and grading of activity in the liver in all groups except for AIH patients. Conclusion: Platelet counts can predict hepatic fibrosis in patients with NASH, AIH, or PBC. Clinicians should pay attention to the grading of liver activity in the use of WFA(+)-M2BP.

## 1. Introduction

Nonalcoholic fatty liver disease (NAFLD) is the most prevalent chronic liver disease. It currently affects up to 30% of the population in the United States and European countries [1]. It represents a serious public health problem all over the world. NAFLD represents a histopathological spectrum ranging from simple steatosis to steatosis with inflammation and/or fibrosis. The latter is called nonalcoholic steatohepatitis (NASH). Patients with NASH have an increased risk of advanced liver fibrosis, cirrhosis, hepatocellular carcinoma (HCC), and liver-related mortality. In general, NASH is diagnosed based on histological findings from a liver biopsy.

Autoimmune hepatitis (AIH) is characterized as chronic inflammation of the liver caused by immune abnormalities. The diagnosis of AIH is based on the existence of autoantibodies, hypergammaglobulinemia, histological findings of interface hepatitis, portal lymphocytic infiltration involving primarily plasma cells [2], and the absence of other causes of chronic hepatitis. Primary biliary cholangitis (PBC) [3], also regarded as one of the autoimmune disorders, is characterized by the existence of anti-mitochondrial antibodies and histological findings of portal inflammatory infiltration, and chronic non-suppurative destructive cholangitis [4]. All of these diseases rely on histological findings for accurate diagnosis, evaluation of staging of fibrosis, and grading of activity. However, the number of people unable to tolerate a liver biopsy is growing with the advancement of an aging society in Japan. Liver biopsy itself also has the risk of sampling errors [5].

Because of these issues, we need innovative non-invasive biomarkers for the diagnosis and evaluation of fibrosis and inflammation. Platelet counts and serum hyaluronic acid levels are reported as good biomarkers of liver fibrosis [6,7]. There are also multiple non-invasive methods based on blood tests for predicting liver fibrosis, especially in patients with chronic hepatitis C, such as the aspartate aminotransferase (AST) to platelet index (APRI) [8] and the fibrosis index based on four factors (FIB-4) [9]. FIB-4 includes four factors: age, alanine aminotransferase (ALT), aspartate aminotransferase (AST), and platelet counts [9]. Transient elastography could also predict liver fibrosis especially in patients with chronic hepatitis C [10].

In the previous report [11], 90K/Mac-2-binding protein (M2BP) is a heavily N-glycosylated glycoprotein, being secreted as a ligand of galectin-3 (Mac-2). Recently, several researchers from Japan have reported that Wisteria floribunda agglutinin-positive human Mac-2-binding protein (WFA(+)-M2BP) is a novel non-invasive method of estimating liver fibrosis [12,13]. M2BP has been shown to have multibranching and sialylated N-glycans. Changes in the quality and quantity of M2BP produced are observed during the progression of fibrosis, and these are induced by changes in N-glycosylation. Kuno et al. reported that a rapid and simple glycan-based immunoassay for WFA(+)-M2BP can quantify fibrosis [14]. There are many reports that support the quality of WFA(+)-M2BP in the assessment of fibrosis in HCV patients [15] and PBC patients [16]. Further study may be needed to validate the diagnostic utility of WFA(+)-M2BP in the diagnosis of fibrosis in the full range of chronic hepatitis patients.

In this article, we examined the utility of several non-invasive biomarkers, especially WFA(+)-M2BP, for the evaluation of the staging of fibrosis and grading of activity in several chronic liver diseases such as NASH, AIH, and PBC patients who were histologically diagnosed at a university hospital located in an urban area of Japan.

## 2. Materials and Methods

### 2.1. Research Subjects

Between 2010 and 2014, ninety-four patients diagnosed with NASH, AIH, and PBC through liver biopsy specimens were studied retrospectively. Patients’ age and gender were recorded. All patients were diagnosed and reviewed by at least three hepatologists at the Department of Gastroenterology and Nephrology, Chiba University, Graduate School of Medicine located in an urban area of Japan. Written informed consent was obtained from all patients before liver biopsy. The study was conducted in accordance with the Declaration of Helsinki, and the protocol was approved by the Ethics Committee of Chiba University School of Medicine (No. 1841).

### 2.2. Liver Biopsy and Evaluation of Biopsy Samples

Liver biopsy was performed with 16–20 gauge automatic Tru-cut biopsy needles under ultrasound guidance from the right lobe of the liver. Mean length of liver biopsies was ~20 mm and included at least two complete portal tract areas. Biopsy samples were immediately placed into 10% formalin solution (Wako Pure Chemical, Osaka, Japan). Three hepatologists blindly evaluated the histopathological findings of the liver biopsy samples after hematoxylin and eosin (H&E) staining. The liver tissue samples were evaluated for fibrosis staging and inflammatory activity grading of the liver [17].

Histological NASH was defined as lobular inflammation, ballooning degeneration with or without pericellular fibrosis, and/or Mallory–Denk bodies in the absence of other liver diseases [18]. A diagnosis of AIH was made based on the presence of anti-nuclear antibodies (ANA) and/or anti-smooth muscle antibodies (ASMA), and on the criteria defined by the International Autoimmune Hepatitis Group (IAIHG) [19]. The diagnosis of PBC was based on established criteria that included a cholesteric serum biochemical liver test, positive testing for antimitochondrial antibodies, and liver biopsy findings [4,20]. We classified samples from NASH, PBC, and AIH patients into: no fibrosis (F0), mild fibrosis (F1), moderate fibrosis (F2), severe fibrosis (F3), or cirrhosis (F4); and minimal activity (A0), mild activity (A1), moderate activity (A2), or severe activity (A3) for staging of fibrosis and grading of activity of the liver, respectively.

### 2.3. Clinical and Biological Data

Serum samples were collected at the time of liver biopsy. Alanine aminotransferase (ALT), AST, bilirubin, albumin, hyaluronic acid, blood cell counts, and other biological data were analyzed at Chiba University hospital. WFA(+)-M2BP was quantified at Sysmex CO (Kobe, Japan). Liver stiffness was assessed using Fibroscan (Echosens, Paris, France) by experienced operators between the right lower ribs. The AST to ALT ratio (AAR) was calculated using the following equation: AAR = AST/ALT. The APRI was calculated using the following equation: APRI = [AST (/35 IU/L)/platelet counts (10^3^/μL)] × 100 [8,21]. FIB-4 was calculated using the following equation: FIB-4 = [AST (IU/L) × Age (years)]/[ALT (IU/L)^1/2^ × platelet counts (10^3^/μL)] [10].

### 2.4. Statistical Analysis

Demographic and clinical differences were tested using Student’s *t*-test and chi-square tests. Pearson correlation analysis was used to explore the association of non-fibrotic factors and staging of fibrosis. For all tests, two-sided *p*-values were calculated and the results were considered statistically significant at *p* < 0.05.

## 3. Results

### 3.1. Patient Characteristics

The baseline demographic and clinical characteristics of patients in this study are summarized in Table 1. AIH (94%) and PBC (83%) were female-dominant, compared with NASH patients (59% female; *p* < 0.01 and *p* < 0.05, respectively). The mean body mass index (BMI) was significantly higher in the NASH group (for both, *p* < 0.01). The levels of AST and ALT were significantly higher among AIH patients. The levels of immunoglobulin G (IgG) were significantly higher in AIH patients compared with those of NASH and PBC patients (*p* = 0.02 and *p* < 0.01). The levels of immunoglobulin M (IgM) were significantly higher in PBC patients compared with those of NASH and AIH patients (for both, *p* < 0.01). We did not observe any differences in immunoglobulin A (IgA) levels between any patient groups.

### 3.2. Diagnostic Performance of Noninvasive Fibrotic Markers in Comparison with Liver Biopsy

**Figure 1:**
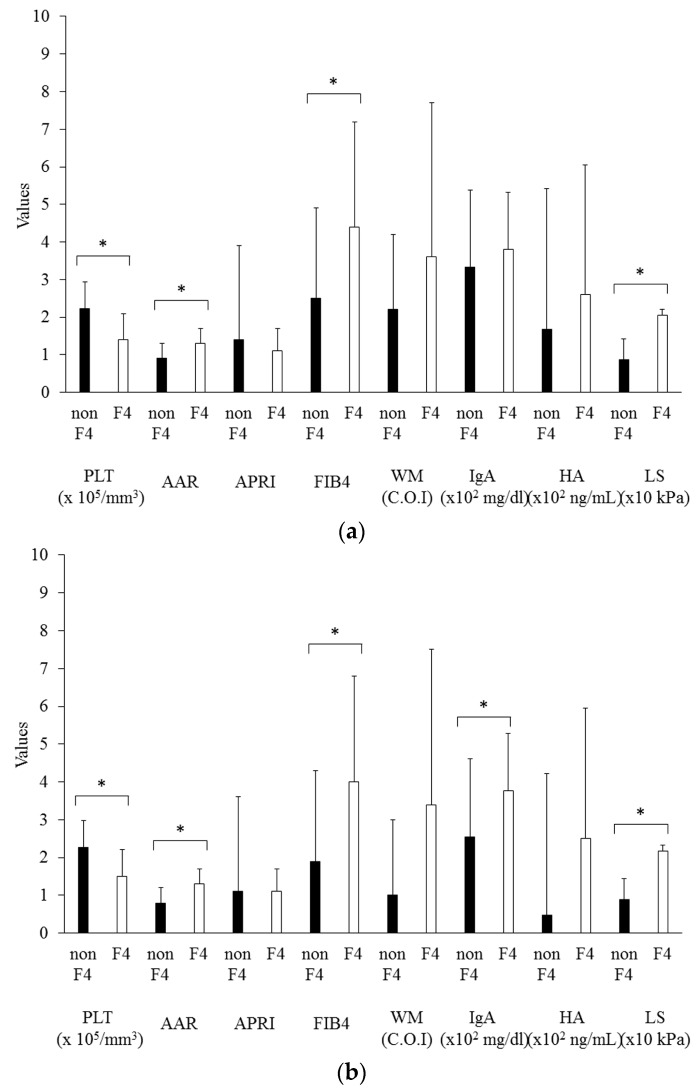

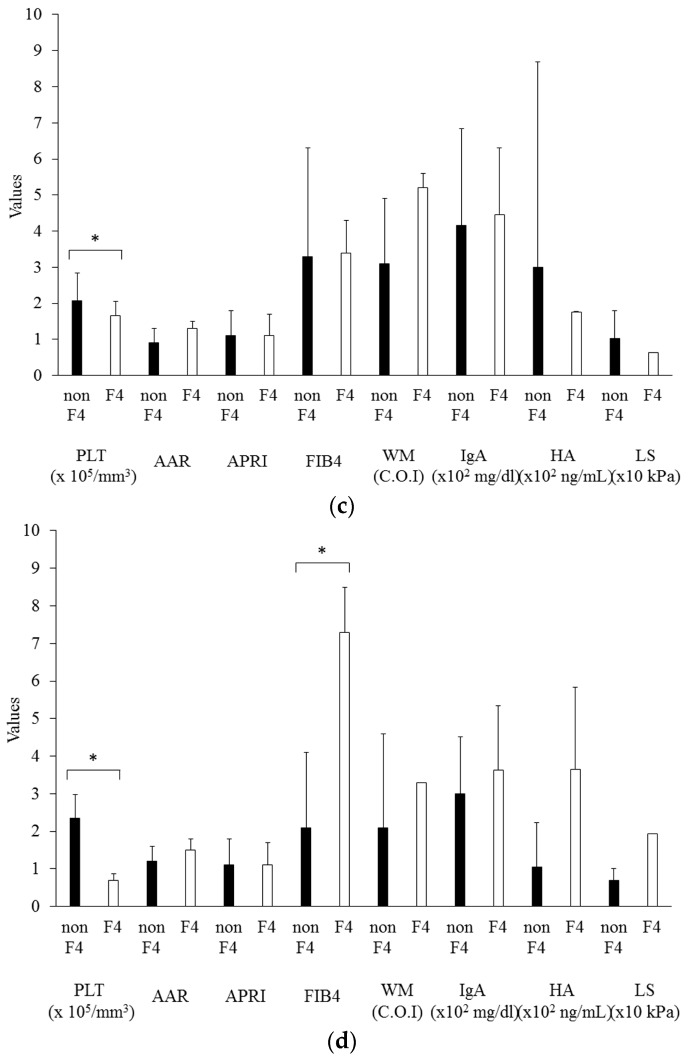
Non-invasive fibrotic markers in non-cirrhotic (non F4) patients and cirrhotic (F4) patients. (**a**) Total patients; (**b**) NASH patients; (**c**) AIH patients; (**d**) PBC patients. The bar graph represents the mean values, and the error bar represents standard deviation values. AAR, AST to ALT ratio; APRI, aspartate aminotransferase to platelet ratio index; FIB4, fibrosis index based on four factors; WM, Wisteria floribunda agglutinin-positive human Mac-2-binding protein; IgA, immunoglobulin A; HA, hyaluronic acid; LS, liver stiffness; * *p* < 0.05.

Serum fibrotic markers can be useful for assessing liver fibrosis although it has several limitations which are affected by liver inflammation. Next, we evaluated the relationship between histological staging of fibrosis (F stage) and grading of activity (A grade). The values of each noninvasive fibrotic marker were significantly increased in patients without cirrhosis (F1/F2/F3) vs patients with cirrhosis (F4), as shown in Figure 1. Only platelet levels were significantly lower in F4 patients among all groups.

### 3.3. The Effects of Hepatic Inflammation on Noninvasive Fibrotic Markers

**Figure 2:**
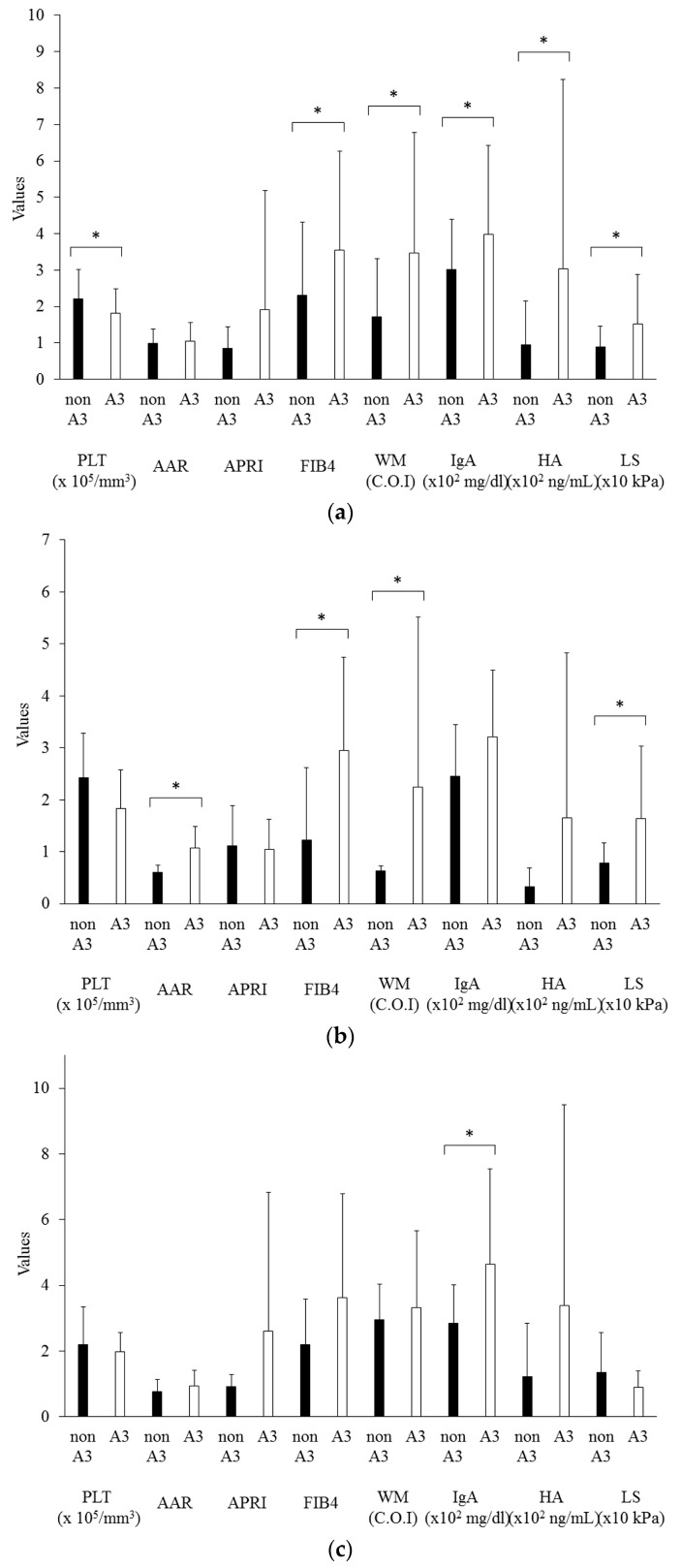

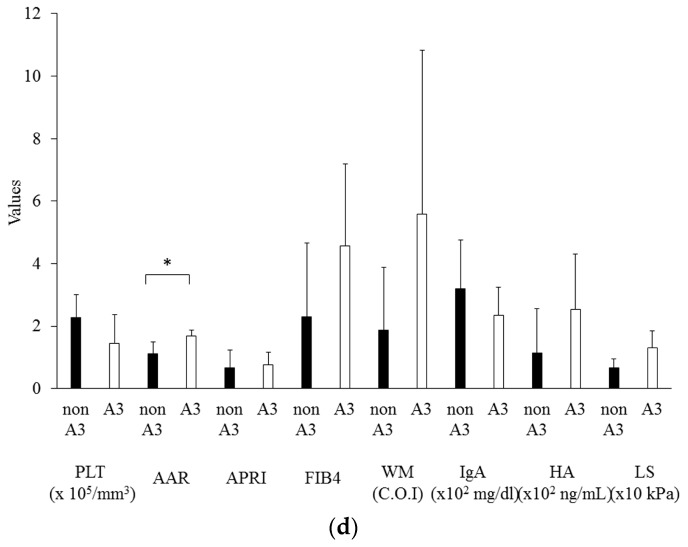
Non-invasive fibrotic markers in patients with mild and moderate inflammation (non A3) and with severe inflammation (A3). (**a**) Total patients; (**b**) NASH patients; (**c**) AIH patients; (**d**) PBC patients. The bar graph represents the mean values, and the error bar represents standard deviation values. AAR, AST to ALT ratio; APRI, aspartate aminotransferase to platelet ratio index; FIB4, fibrosis index based on four factors; WM, Wisteria floribunda agglutinin-positive human Mac-2-binding protein; IgA, immunoglobulin A; HA, hyaluronic acid; LS, liver stiffness; * *p* < 0.05.

The values of several noninvasive fibrotic markers were also significantly increased in patients with mild and moderate inflammation (A1 and A2) vs marked inflammation (A3), as shown in Figure 2. Among NASH patients, AAR, FIB-4, WFA(+)-M2BP, and liver stiffness were significantly higher in patients with marked inflammation. IgA was the only marker that was significantly higher among AIH patients with marked inflammation. AAR was the only marker that was significantly higher among PBC patients with marked inflammation. The relationship between histological F stage and A grade was also evaluated. Each group except for AIH patients had a significant correlation between these two parameters (Table 2).

## 4. Discussion

The present study demonstrates that the serum platelet counts are significantly correlated with the histological staging of fibrosis in NASH patients (Figure 1). We recommend the use of platelet counts rather than serum WFA(+)-M2BP levels in the prediction of liver fibrosis in NASH, PBC, and AIH patients (Figure 1). Previous studies [12,13,14,15,16] have tried to show the effectiveness of this novel non-invasive biomarker for the diagnosis and evaluation of fibrosis and inflammation in various chronic liver diseases. In chronic hepatitis C patients, several studies have reported the effectiveness of several non-invasive biomarkers such as platelet levels, ferritin, and hyaluronic acid [8,9]. In addition to these biomarkers, the efficacy of several fibrosis scoring systems such as the FIB-4 index are reported [9].

The noninvasive markers that have significant correlation with staging of fibrosis (F stage) among NASH patients are platelet counts, AAR, FIB-4, WFA(+)-M2BP, IgA, and liver stiffness. Despite the relatively small sample size, NAFLD fibrosis score (NFS) of NASH patients in the present study was 1.257 ± 1.696, and the NFS of 2, 12, and 20 patients was <−1.455 (predictor of stage F0–F2), ≤−1.455 to ≤0.675 (intermediate score), and >0.675 (stage F3–F4), respectively [22]. Platelet counts were the only marker that had significant correlation with F stage among AIH patients. Platelet counts and FIB-4 showed significant correlation with F stage among PBC patients (Figure 1). Platelet counts were reduced in accordance with the progression of liver disease in chronic HCV infection [10]. In the present study, we also showed that the level of platelet counts is a useful predictor of hepatic fibrosis progression in patients with NASH, AIH, and PBC. 

The noninvasive markers that have significant correlation with A grade were also examined (Figure 2). AAR, FIB-4, WFA(+)-M2BP, and liver stiffness were significantly correlated with A grade among NASH patients. IgA was the only marker that had significant correlation with A grade among AIH patients, although IgG did not have significant correlation with A grade among any groups (data not shown). AAR showed significant correlation with A grade among PBC patients. Clinicians should also pay attention to the grading of inflammatory activity of the liver in the use of WFA(+)-M2BP.

Elevated serum levels of M2BP have been found in several human diseases, including autoimmune diseases [23], cancer [24], and hepatitis B virus (HBV) [24], HCV [24], or human immunodeficiency virus (HIV) [25]. M2BP may play a role in the immunological reactions related to these diseases [2]. Grassadonia et al. [24] reported that M2BP is a highly glycosylated secreted protein, extensively studied in human cancer, which binds galectin-1, galectin-3, and galectin-7. High expression levels of M2BP are associated with shorter survival, the occurrence of metastases or reduced response to chemotherapy in patients with cancers [24]. Serum M2BP levels are increased in chronic HCV infection to levels significantly higher than in chronic HBV infection [25]. M2BP is associated with the disease progression in HIV-infected individuals [26]. Further studies are needed to elucidate the mechanism of M2BP and WFA(+)-M2BP elevation in advanced liver diseases.

## 5. Conclusions

Kamada et al. [27,28] reported that serum M2BP level is a useful diagnostic biomarker for the prediction of disease severity in NASH. As the present study does not include the patients with obvious cirrhosis, in whom liver biopsy cannot be performed, we cannot examine the effects of damage of liver functions well. Further studies will be needed. In conclusion, platelet counts may be predictive of the hepatic fibrosis in patients with NASH. 

## Figures and Tables

**Table 1:** Baseline characteristics of 94 patients with nonalcoholic steatohepatitis (NASH), autoimmune hepatitis (AIH), or primary biliary cholangitis (PBC) at liver biopsy.

	Total Patients	NASH	AIH	PBC
*n* (Male/Female)	94 (21/73)	34 (14/20)	31 (2/29)	29 (5/24)
Age	58.0 ± 13.6	53.9 ± 16.4	60.1 ± 11.0	60.5 ± 11.4
BMI (kg/m^2^)	25.0 ± 4.4	27.9 ± 3.7	22.9 ± 3.6	23.9 ± 4.1
A grade (A1/A2/A3)	17/34/37	6/14/12	1/6/21	10/14/4
F stage (F1/F2/F3/F4)	42/10/16/21	14/2/1/16	12/4/10/2	16/4/5/3
Platelet counts (×10^4^/mm^3^)	20.2 ± 7.9	19.1 ± 8.1	20.0 ± 7.9	21.9 ± 8.0
AST (IU/L)	82.2 ± 141.5	66.0 ± 39.0	139.4 ± 233.8	40.1 ± 19.0
ALT (IU/L)	98.6 ± 154.8	84.1 ± 62.6	170.6 ± 245.4	38.4 ± 27.2
ALP (IU/L)	381.8 ± 232.0	301.4 ± 155.3	369.6 ± 154.7	488.9 ± 323.8
ALB (g/dL)	4.1 ± 0.4	4.3 ± 0.4	3.9 ± 0.4	4.0 ± 0.4
T-Chol (mg/dL)	186.2 ± 43.9	176.4 ± 45.9	182.1 ± 41.5	203.4 ± 40.5
LDL-Chol (mg/dL)	110.6 ± 36.7	106.7 ± 34.1	109.3 ± 41.0	117.7 ± 34.8
TG (mg/dL)	133.1 ± 66.6	152.8 ± 83.8	118.7 ± 45.8	124.7 ± 58.1
HbA1c (%)	5.2 ± 0.7	5.6 ± 0.7	5.1 ± 0.7	5.0 ± 0.5
Ferritin (ng/mL)	308.4 ± 520.3	346.1 ± 454.0	459.4 ± 724.6	95.9 ± 98.6
ANA (-fold)	325.7 ± 434.7	56.5 ± 63.9	457.3 ± 462.8	518.5 ± 506.5
IgG (mg/dL)	1864.8 ± 902.1	1390.0 ± 347.0	2423.5 ± 1243.4	1824.2 ± 532.2
IgA (mg/dL)	344.8 ± 195.7	310.9 ± 128.5	413.5 ± 268.2	311.1 ± 151.2
IgM (mg/dL)	187.5 ± 131.7	121.4 ± 55.9	165.3 ± 111.3	288.6 ± 155.6

ALB, Albumin; ALP, alkaline phosphatase; ALT, alanine aminotransferase; ANA, anti-nuclear antibody; A grade, grading of activity; AST, aspartate aminotransferase; BMI, body mass index; F stage, staging of fibrosis; HbA1c, hemoglobin A1c; IgA, immunoglobulin A; IgG, immunoglobulin G; IgM, immunoglobulin M; LDL-Chol, low-density lipoprotein cholesterol; T-Chol, total cholesterol; TG, triglyceride.

**Table 2:** Relationships between histological staging of fibrosis (F stage) versus grading of activity (A grade) in patients with nonalcoholic steatohepatitis (NASH), autoimmune hepatitis (AIH), or primary biliary cholangitis (PBC). Pearson correlation coefficient between F stage and A grade was indicated by *r*.

	*r*	*P*-Value
NASH	0.65	<0.01
AIH	0.30	0.12
PBC	0.64	<0.01
